# Coronavirus Disease-2019: Knowledge, Attitude, and Practices of Health Care Workers at Makerere University Teaching Hospitals, Uganda

**DOI:** 10.3389/fpubh.2020.00181

**Published:** 2020-04-30

**Authors:** Ronald Olum, Gaudencia Chekwech, Godfrey Wekha, Dianah Rhoda Nassozi, Felix Bongomin

**Affiliations:** ^1^College of Health Sciences, Makerere University, Kampala, Uganda; ^2^Department of Medicine, College of Health Sciences, Makerere University, Kampala, Uganda; ^3^Department of Medical Microbiology and Immunology, Faculty of Medicine, Gulu University, Gulu, Uganda

**Keywords:** COVID-19, Uganda, KAPs, healthcare workers, Makerere University Teaching Hospitals

## Abstract

**Background:** Coronavirus disease-2019 (COVID-19) is an emerging public health problem threatening the life of over 2.4 million people globally. The present study sought to determine knowledge, attitude and practices (KAP) of health care workers (HCWs) toward COVID-19 in Makerere University Teaching Hospitals (MUTHs) in Uganda.

**Methods:** An online cross sectional, descriptive study was undertaken through WhatsApp Messenger among HCWs in four MUTHs. HCWs aged 18 years and above constituted the study population. KAP toward COVID-19 was assessed by using a pre-validated questionnaire. Bloom's cut-off of 80% was used to determine sufficient knowledge (≥80%), positive attitude (≥4), and good practice (≥2.4). All analyses were performed using STATA 15.1 and GraphPad Prism 8.3.

**Results:** Of the 581 HCWs approached, 136 (23%) responded. A vast majority of the participants were male (*n* = 87, *n* = 64%), with a median age of 32 (range: 20–66) years. Eighty-four (62%) were medical doctors and 125 (92%) had at least a bachelor's degree. Overall, 69% (*n* = 94) had sufficient knowledge, 21% (*n* = 29) had positive attitude, and 74% (*n* = 101) had good practices toward COVID-19. Factors associated with knowledge were age >40 years (aOR: 0.3; 95% CI: 0.1–1.0; *p* = 0.047) and news media (aOR: 4.8; 95% CI: 1.4–17.0; *p* = 0.015). Factors associated with good practices were age 40 years or more (aOR: 48.4; 95% CI: 3.1–742.9; *p* = 0.005) and holding a diploma (aOR: 18.4; 95% CI: 1–322.9; *p* = 0.046).

**Conclusions:** Continued professional education is advised among HCWs in Uganda to improve knowledge of HCWs hence averting negative attitudes and promoting positive preventive and therapeutic practices. We recommend follow up studies involving teaching and non-teaching hospitals across the country.

## Introduction

Coronavirus Disease 2019 also known as COVID-19 is a rapidly expanding pandemic caused by a novel human coronavirus (SARS-COV-2) previously known as 2019-nCov (1, 2). COVID-19 was first reported in December 2019 among patients with viral pneumonia symptoms in Wuhan, China (3, 4). They were found to be related with the Huanan seafood market in Wuhan, in the Hubei province of China, where other non-aquatic animals were also being sold before the outbreak (5). As of 20th April 2020, over 2.4 million cases and 165,000 deaths have been reported globally (6, 7). Europe is the most affected with over 50% of cases and 60% of deaths reported in this region (8). United States of America has the highest number of cases globally (695,350 cases) and the highest number of deaths (32,427 deaths) (8). African region is the least affected with 13,892 cases and 628 deaths, but the numbers are increasing (8). Uganda has so far confirmed 55 cases of COVID-19 as of 20th April 2020 (7, 8).

SARS-COV-2 is transmitted from person-to-person through inhalation of aerosols from an infected individual (3). Old age and patients with pre-existing illnesses (like hypertension, cardiac disease, lung disease, cancer, or diabetes) have been identified as potential risk factors for severe disease and mortality (9, 10). To date, there is no antiviral curative treatment or vaccine that has been recommended for COVID-19 (11). More information about its distribution, transmission, pathophysiology, treatment, and prevention are being studied. World Health Organization (WHO) recommends prevention of human-to-human transmission by protecting close contacts and health care workers from being infected and stopping infections from animal sources (8). Primary preventive measures include regular hand washing, social distancing, and respiratory hygiene (covering mouth and nose while coughing or sneezing) (12, 13).

Healthcare workers (HCWs) are at the frontline of COVID-19 pandemic response and are exposed to dangers like pathogen exposure, long working hours, psychological distress, fatigue, occupational burnout and stigma, and physical violence (14). A poor understanding of the disease among HCWs can result in delayed identification and treatment leading to rapid spread of infections. Over 100 health workers have lost their lives to COVID−19, a tragedy to the world and a barrier to fight against the disease (15). Guidelines for healthcare workers and online refresher courses have been developed by WHO, CDC, and various governmental organizations in various countries to boost the knowledge and prevention strategies (16). There is paucity of literature on KAPs of HCWs toward the COVID-19 pandemic. However, a study with majorly Asian HCWs and medical students revealed that they had insufficient knowledge about COVID-19 but had a positive attitude toward prevention of COVID-19 transmission (17). To our knowledge, no study has been done in sub-Saharan Africa to assess KAPs toward COVID-19 specifically among HCWs. The purpose of the study was to assess the KAPs of HCWs in Uganda toward COVID-19.

## Methodology

### Study Design and Site

An online cross-sectional study was conducted in the first week of April 2020 at four (4) teaching hospitals of Makerere University College of Health Sciences (MakCHS), Makerere University, Kampala, Uganda, i.e., Mulago National Referral Hospital, Mulago Specialized Women and Neonatal Hospital, Kiruddu National Referral Hospital (Directorate of Medicine), and Kawempe National Referral Hospital (Directorate of Obstetrics and Gynecology). The hospitals are components of Mulago Hospital Complex, the largest public hospital in Uganda. The total bed capacities of these hospitals are estimated at 1,800 as of April 2020. There are ~1,300–1,500 healthcare workers.

### Study Population

HCWs (nurses, midwives, internship doctors, medical officers, senior house officers, and specialists) practicing in any of the four MakCHS teaching hospitals who were aged 18 years and above were included in the study after an informed consent. HCWs who were too ill to participate were excluded.

### Study Procedure

Due to the country's lockdown at the time of data collection, we opted to use WhatsApp Messenger (Facebook, Inc., California, USA) for enrolling potential participants. We identified all the existing HCWs WhatsApp groups of the four MaKCHS teaching hospitals. A total of 581 HCWs who were members in the several WhatsApp groups were approached to participate in the study. An online data collection tool was designed and executed using Google Forms (via docs.google.com/forms). The Google Form link to the questionnaire was sent to the enrolled participants via the identified WhatsApp groups.

### Operational Definition

HCWs are defined as all people engaged in activities whose primary intention is to improve health (18). For the purpose of this study, healthcare professionals in primary contact with patients were enrolled. These included nurses, midwives, intern doctors, medical officers, senior house officers, and specialists.

### Study Variables

#### Independent Variables

Demographic details which include sex, age, academic qualification, highest level of education, and sources of information on COVID-19.

#### Dependent Variables

Knowledge, attitude and practices toward COVID-19.

*Knowledge* was assessed using a 11-item questionnaire adapted from Zhong et al. (19) and modified to suit HCWs, each correct answer weighing one point. The questions were about clinical presentations, transmission, prevention and control of COVID-19. Each correct response weight 1 point and 0 for incorrect responses. The higher the points, the more knowledgeable the HCW is.

*Attitudes* were assessed using 5 Likert-item questions that have been adopted from Goni et al. (20) and modified appropriately for COVID-19 by the authors. The responses were; strongly disagree, disagree, neutral, agree and strongly agree each weighing 1–5 respectively for each positive statement. Some questions were reversed to eliminate biases of giving a single similar response in all the items.

*Practices* were assessed using five Likert-item questions that have been developed from the WHO and Ministry of Health Uganda recommended practices for prevention of COVID-19 transmission i.e., hand washing, avoiding crowded places, keeping social distance (1 meter apart), avoiding touching of face, and avoiding handshakes. The responses were; always, occasional, and never each weighing 3, 2, and 1 point respectively for a good practice. The link to the questionnaire can be accessed in the Supplementary Material section below.

### Data Management and Analyses

Fully completed questionnaires were extracted from Google Forms and exported to a Microsoft Excel 2016 for cleaning and coding. The cleaned data was exported to STATA version 15.1 and GraphPad 8.3 for analyses. Numerical data was summarized as means and standard deviations or median and range as appropriate. Categorical data was summarized as frequencies and proportions. Bloom's cut-off of 80% was used to determine sufficient knowledge (≥80%), positive attitude (≥4), and good practice (≥2.4) (21). Associations between independent variables and dependent variables were assessed using multivariate analysis in STATA 15.1 software. Kruskalis-Wallis and One-Way Analysis of Variance using GraphPad Prism 8.3 was done to compare KAPs across groups. A *p* < 0.05 is considered statistically significant. The data set can be accessed via a link provided in the Supplementary Materials section.

## Results

Of the 581 HCWs approached, total of 136 HCWs responded (response rate = 23%). A vast majority of the participants were male (*n* = 87, 64%), with a mean age of 34 (SD: 7.9) years and below 40 years of age (*n* = 107, 79%). Majority of the participants were practicing in Mulago National Referral Hospital (*n* = 73, 54%) and a minority in Mulago Women and Neonatal Hospital (*n* = 8, 6%). Eighty-four (62%) participants were medical doctors, 15 (11%) were nurses, and 7 (5) were midwives. Of the 136 participants, 125 (92%) had at least a bachelor's degree and a minority had an ordinary diploma or a certificate. The main sources of information about COVID-19 among participants were information from international health organizations like the CDC and WHO, Ministry of Health, Uganda media sites, News Media and social media such as WhatsApp and Facebook. Table 1 summarizes the sociodemographic characteristics of the participants.

**Table 1:** Sociodemographic characteristics of the participants.

**Variable**	**Freq (*n*)**	**%**
**Sex**		
Male	87	64
Female	49	36
**Age (Mean, SD)**	34.0	7.9
18–39	107	79
≥40	29	21
**Place of work**		
Mulago National Referral Hospital	73	54
Kiruddu National Referral Hospital	41	30
Kawempe National Referral Hospital	14	10
Mulago Specialized Women and Neonatal Hospital.	8	6
**Qualification**		
Senior house officer	48	35
Specialist	30	22
Medical officer	21	15
Intern doctor	15	11
Nurse	15	11
Midwife	7	5
**Highest level of education**		
Bachelors	75	55
Masters	43	32
Diploma	8	6
PhD	7	5
Certificate	3	2
**Source of information on COVID-19**		
International health organization e.g., WHO	119	88
Government sites and media e.g., MoH-Uganda	107	79
Social media e.g., WhatsApp, Facebook	100	74
News media e.g., TV, radio, newspaper	98	72
Journals	63	46
Others	23	17

*MoH, Ministry of Health; TV, Television*.

### Knowledge

**Figure 1:**
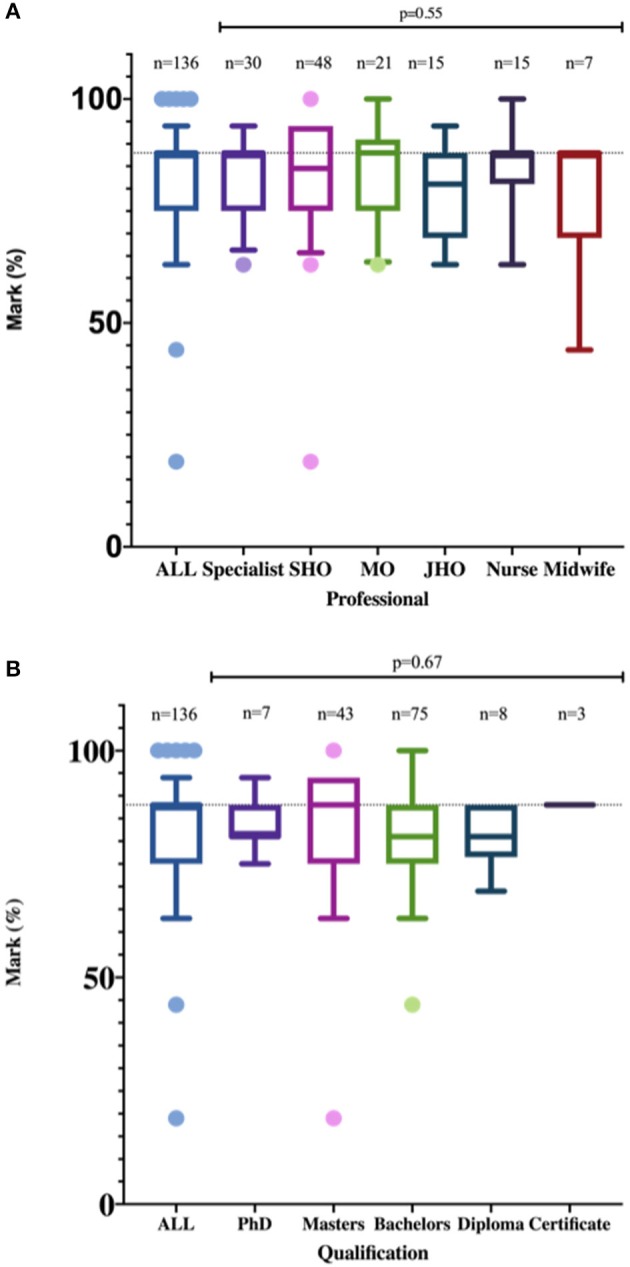
Level of Knowledge of healthcare workers stratified by profession **(A)** and qualifications **(B)**. There was no statistically significant difference in the level of knowledge about COVID-19 among health care workers in Uganda irrespective of their professions or qualifications.

The mean knowledge score was 82.4 (SD: 11.2) percent. Sixty-nine percent (*n* = 94) of the participants scored 80% or more and were considered to have sufficient knowledge. Only two participants scored below 50%. Factors associated with knowledge were age >40 (aOR: 0.3; 95% CI: 0.1–1.0; *p* = 0.047) and news media (aOR: 4.8; 95% CI: 1.4–17.0; *p* = 0.015, **Table 4**). The mean knowledge score of male participants was higher than those of female participants (83.2 vs. 80.9%), However this difference was not statistically significant (*p* = 0.38). The level of knowledge among the healthcare workers were similar irrespective of the cadre (*p* = 0.55) or academic qualifications (*p* = 0.67, Figure 1).

### Attitude

The mean attitude score 3.4 (SD: 0.6). Overall, there was poor attitude among HCWs toward COVID-19. Only 21% (*n* = 29) of the participants had a good attitude toward COVID-19. Of these, 59% (*n* = 17) and 62% (*n* = 18) were males and from Mulago National Referral Hospital, respectively. Ten percent (*n* = 13) reported that black race is protective against COVID-19 and only 44% (*n* = 60) were confident that they would participate in the management of a patient with COVID-19 (Table 2). When asked about the preparedness of Uganda, up to 29% (*n* = 40) believed that Uganda was not in a good position to contain the COVID-19 pandemic. Table 2 shows the frequency of the responses and Table 3 shows the mean attitude scores and percentage of HCWs with good attitude. In Table 4, multivariate analysis revealed that HCWs who used mainstream media like television to access information on COVID-19 were four times more likely to have a good attitude, this was however not statistically significant (aOR = 3.7, 95% CI = 0.8–16.8, *p* = 0.085). There was no statistically significant correlation between attitude and the sociodemographic variable (sex, age, hospital, qualification, and level of education) at (*p* < *0.05*, Table 4.)

**Table 2:** Knowledge, attitude and practices of healthcare workers at Makerere University Teaching Hospitals toward COVID-19.

**Question**	**Responses (*****N*** **=** **136)**		
**Knowledge**	**True** **(*n*, %)**	**False** **(*n*, %)**	**I don't know** **(*n*, %)**
**The main clinical symptoms of COVID-19 are; (Tick all that apply)**			
Cough	126 (93)	-	-
Fever	133 (98)	-	-
Sore throat	118 (87)	-	-
Runny nose	99 (72)	-	-
Myalgia (muscle pain)	74 (54)	-	-
Diarrhea	47 (35)	-	-
There is currently no effective cure for COVID-19, but early symptomatic and supportive treatment can help most patients recover from the infection (true).	131 (96)	3 (2)	2 (1)
Not all persons with COVID-19 will develop severe cases. Only those who are elderly, have chronic illnesses, and are obese are more likely to be severe cases (true).	109 (80)	25 (18)	2 (1)
Eating or contacting wild animals would result in the infection by the COVID-19 virus (false).	50 (37)	65 (48)	21 (15)
Persons with COVID-2019 cannot transmit the virus to others when a fever is not present (false).	6 (4)	128 (94)	2 (1)
The COVID-19 virus spreads via respiratory droplets of infected individuals (true).	132 (97)	3 (2)	1 (1)
Wearing general medical masks can prevent one from acquiring infection by the COVID-19 virus (true).	75 (55)	56 (41)	5 (4)
It is not necessary for children and young adults to take measures to prevent the infection by the COVID-19 virus (false).	5 (4)	131 (96)	0 (0)
To prevent the infection by COVID-19, individuals should avoid going to crowded places such as bus parks and avoid taking public transportations (true).	133 (98)	1 (1)	2 (1)
Isolation and treatment of people who are infected with the COVID-19 virus are effective ways to reduce the spread of the virus (true).	134 (99)	1 (1)	1 (1)
People who have contact with someone infected with the COVID-19 virus should be immediately isolated in a proper place. In general, the observation period is 14 days (true).	136 (100)	0 (0)	0 (0)
**Practice**	**Always** **(*****n*****, %)**	**Occasional** **(*****n*****, %)**	**Never** **(*****n*****, %)**
In recent days, have you gone to any crowded place?	11 (8)	68 (50)	57 (42)
In recent days, I have worn a mask when in contact with patients?	74 (54)	53 (39)	9 (7)
In the recent days, I have refrained from shaking hands.	113 (83)	22 (16)	1 (1)
In the recent days, I have washed my hands before and after handling each patient?	100 (74)	30 (22)	6 (4)
In the recent days, I have avoided patients with signs and symptoms suggestive of COVID-19.	39 (29)	42 (31)	55 (40)

**Table:** 

**Attitude**	***SD*** **(*****n*****, %)**	***D*** **(*****n*****, %)**	***N*** **(*****n*****, %)**	***A*** **(*****n*****, %)**	***SA*** **(*****n*****, %)**
Black race is protective toward COVID-19 disease.	50 (37)	34 (25)	39 (29)	9 (7)	4 (3)
Wearing a well-fitting face mask is effective in preventing COVID-19.	13 (10)	10 (7)	10 (7)	75 (55)	28 (21)
Using a hand wash can prevent you from getting COVID-19.	10 (7)	5 (4)	2 (1)	72 (53)	47 (35)
When a patient has signs and symptoms of COVID-19, I can confidently participate in the management of the patient.	25 (18)	23 (17)	28 (21)	48 (35)	12 (9)
Uganda is in a good position to contain COVID-19.	31 (23)	35 (26)	30 (22)	34 (25)	6 (4)

*SD, Strongly Disagree; D, Disagree; N, Neutral; A, Agree; SA, Strongly Agree*.

**Table 3:** Knowledge, attitude and practices among healthcare workers at Makerere University Teaching Hospitals.

**Variable**	**Knowledge** **(% score)**	**Sufficient knowledge** **(*n* = 94)**	**Attitude** **(Max = 5)**	**Positive attitude** **(*n* = 29)**	**Practice** **(Max = 3)**	**Good practice** **(*n* = 101)**
	**Mean ± SD**	**Freq (%)**	**Mean ± SD**	**Freq (%)**	**Mean ± SD**	**Freq (%)**
**Overall**	82.4 ± 11.2	94 (69)	3.4 ± 0.6	29 (21)	2.5 ± 0.3	101 (74)
**Sex**						
Male	83.2 ± 10	61 (65)	3.4 ± 0.7	17 (59)	2.5 ± 0.3	68 (67)
Female	80.9 ± 13	33 (35)	3.5 ± 0.6	12 (41)	2.5 ± 0.4	33 (33)
**Place of work**						
Mulago National Referral Hospital	82.4 ± 10.5	48 (51)	3.4 ± 0.7	18 (62)	2.5 ± 0.3	54 (53)
Kiruddu National Referral Hospital	82.2 ± 13.5	30 (32)	3.4 ± 0.6	6 (21)	2.6 ± 0.3	32 (32)
Kawempe National Referral Hospital	81.7 ± 8.3	10 (11)	3.4 ± 0.6	2 (7)	2.4 ± 0.4	8 (8)
Mulago Specialized Women and Neonatal Hospital	84.4 ± 9.4	6 (6)	3.6 ± 0.7	3 (10)	2.5 ± 0.3	7 (7)
**Qualification**						
Senior house officer	82.3 ± 12.9	33 (35)	3.4 ± 0.7	11 (38)	2.5 ± 0.4	35 (35)
Specialist	83.1 ± 8.6	21 (22)	3.5 ± 0.6	8 (28)	2.6 ± 0.3	26 (26)
Medical officer	83.9 ± 10.7	14 (15)	3.6 ± 0.6	4 (14)	2.5 ± 0.3	14 (14)
Nurse	85 ± 8.1	14 (15)	3.3 ± 0.7	2 (7)	2.5 ± 0.4	12 (12)
Intern doctor	78.8 ± 10	8 (9)	3.3 ± 0.7	2 (7)	2.3 ± 0.2	9 (9)
Midwife	76.8 ± 16.4	4 (4)	3.5 ± 0.6	2 (7)	2.5 ± 0.3	5 (5)
**Highest level of education**						
Bachelors	81.9 ± 10.7	48 (51)	3.4 ± 0.7	17 (59)	2.4 ± 0.3	51 (50)
Masters	82.7 ± 13.6	31 (33)	3.5 ± 0.6	9 (31)	2.5 ± 0.3	33 (33)
PhD	83.9 ± 6.1	6 (6)	3.3 ± 1	2 (7)	2.7 ± 0.1	7 (7)
Diploma	81.3 ± 6.7	6 (6)	3.5 ± 0.5	1 (3)	2.6 ± 0.3	7 (7)
Certificate	87.5 ± 0	3 (3)	3.3 ± 0.2	0 (0)	2.8 ± 0.2	3 (3)
**Source of information on COVID-19**						
International health organizations e.g., WHO	82.7 ± 11.4	85 (90)	3.4 ± 0.7	25 (86)	2.5 ± 0.3	90 (89)
Government sites and media	83.6 ± 9	77 (82)	3.5 ± 0.6	21 (72)	2.5 ± 0.3	83 (82)
News media e.g., TV, radio, newspaper	84.6 ± 8.3	76 (81)	3.5 ± 0.6	23 (79)	2.5 ± 0.3	77 (76)
Social media e.g., WhatsApp, Facebook	84.1 ± 8.8	73 (78)	3.5 ± 0.6	23 (79)	2.5 ± 0.3	78 (77)
Journals	83.4 ± 9.1	43 (46)	3.4 ± 0.7	12 (41)	2.5 ± 0.3	50 (50)
Others	80.2 ± 15.4	15 (16)	3.3 ± 0.8	4 (14)	2.6 ± 0.3	18 (18)

**Table 4:** Factors associated with knowledge, attitude and practices among healthcare workers at Makerere University Teaching Hospitals.

**Variable**	**Knowledge**	**Attitude**	**Practices**
	**aOR (95% CI)**	**aOR (95% CI)**	**aOR (95% CI)**
**Sex**			
Male	1	1	1
Female	1.1 (0.4–3)	1.4 (0.5–3.8)	0.4 (0.1–1.2)
**Age**			
18–39	1	1	1
≥40	0.3 (0.1–1)^*^	1.3 (0.4–4.4)	48.4 (3.1–742.9)^*^
**Place of work**			
Mulago National Referral Hospital	1	1	1
Kiruddu National Referral Hospital	1.2 (0.4–3.4)	0.6 (0.2–1.8)	0.7 (0.2–2.2)
Kawempe National Referral Hospital	2.1 (0.4–10.4)	0.4 (0.1–2.3)	0.6 (0.1–2.8)
Mulago Specialized Women and Neonatal Hospital	1.4 (0.2–11)	1.2 (0.2–7.2)	1.6 (0.1–24.1)
**Qualification**			
Senior house officer	1	1	1
Specialist	1.3 (0.3–6.6)	1.4 (0.3–7.4)	1.7 (0.3–10.4)
Medical officer	2.1 (0.5–8.2)	0.7 (0.1–3.4)	0.5 (0.1–2)
Nurse	3.6 (0.3–51.8)	0.5 (0.1–3.6)	0.5 (0.1–4.1)
Intern doctor	0.5 (0.1–2.2)	0.3 (0–2.1)	0.6 (0.1–2.8)
Midwife	0.7 (0.1–7.6)	1.4 (0.1–13.9)	0.3 (0–3.6)
**Highest level of education**			
Bachelors	1	1	1
Masters	1.8 (0.5–6.5)	0.4 (0.1–1.8)	0.9 (0.2–3.6)
PhD	6.2 (0.4–104.1)	0.5 (0–5.4)	
Diploma	1.03 (0.1–17.7)	0.2 (0–3)	18.4 (1–322.9)^*^
**Source of information on COVID-19**			
International health organization e.g., WHO	2.7 (0.7–10.7)	1.1 (0.2–5.3)	2.7 (0.5–13.8)
Government sites and media e.g., MoH	0.7 (0.2–2.6)	0.3 (0.1–1.3)	1.6 (0.4–6.2)
News media e.g., TV, radio, newspaper	4.8 (1.4–17)^*^	3.7 (0.8–16.8)	0.5 (0.1–2.3)
Social media e.g., WhatsApp, Facebook	0.9 (0.3–2.8)	1.2 (0.3–4.5)	3.5 (1–12.5)
Journals	0.8 (0.3–2.2)	0.5 (0.2–1.5)	2.4 (0.8–7.2)
Others	0.7 (0.2–2.2)	0.9 (0.2–3.5)	0.9 (0.2–3.4)

aOR, adjusted odds ratio;

* *significant at p < 0.05*.

### Practices

Some 54% (*n* = 74) of the HCWs always wore a mask when coming into contact with the patients and up to 96% (*n* = 130) washed their hands before and after touching each patient. Unfortunately, as high as 60% (*n* = 81) of the participants had avoided patients with symptoms similar to those of COVID-19 (Table 2). Overall, up to 74% (*n* = 101) of the participants had good practices (mean score ≥ 2.4, Table 3). Age ≥ 40 and HCWs with diploma were significantly (*p* < 0.05) more likely to have good practices (Table 4).

## Discussion

COVID-19 is an emerging, rapidly changing global health challenge affecting all sectors (22, 23). HCWs are not only at the forefront of the fight against this highly contagious infectious disease but are also directly or indirectly affected by it and the likelihood of acquiring this disease is higher among HCWs compared to the general population (15). It is therefore of paramount importance that HCWs across the world have adequate knowledge about all aspects of the disease from clinical manifestation, diagnosis, proposed treatment, and established prevention strategies.

To the best of our knowledge, this is the first study in Uganda and the sub-Saharan Africa to assess the KAPs of HCWs toward COVID-19. There are also very limited studies that document KAPs among HCWs globally. In the present study, we were able to demonstrate that about seven in 10 of the HCWs had sufficient knowledge about COVID- 19. Among these HCWs, the level of knowledge about COVID-19 was similar irrespective of the age, sex, academic qualification or profession of the HCW.

From our study, a mean knowledge score of 82.4% was obtained on questions about knowledge indicating good knowledge among HCWs at MaKCHS Teaching Hospitals. However, this score is much lower than that reported in Chinese general population (90%) (19) but slightly higher than the KAP toward COVID-19 among US residents (80%) (24). This is possibly because the Chinese and the US studies assessed COVID-19 symptoms using one direct question rather than asking the participants to choose from multiple options. Generally, majority of the HCWs had sufficient knowledge about COVID-19 which is in line with findings in Vietnam about COVID-19 (25). In contrast, it is conflicting to surveys by Bhagavathula et al. on COVID-19 (17), a baseline study among nurses in Gabon on Ebola (26) and HCWs in Ethiopia on Ebola (27) who all reported poor knowledge. From our study, 69% of HCW had sufficient knowledge about COVID-19 which is lower than values reported by Huynh et al. where 88.4% had sufficient knowledge on COVID-19 (25). Further education and training through continuous professional education and journal clubs, particularly on symptoms and transmission are essential in improving the knowledge of HCW about COVID-19 in our setting.

In our study, most of the participants used information from international and governmental media (websites and verified social media pages). Our study suggests that knowledge on COVID-19 was significant among HCWs who used news media such as televisions. This suggests that such media should be frequently used to disseminate information on COVID-19 by the stakeholders. Younger HCW (<40 years) were more likely to have knowledge about COVID-19 unlike in Vietnam where age did not predict knowledge (25). This age difference may be partly due to the diversity of the sources of information frequently used by younger HCW.

About 17% of HCW believed that wearing general medical masks was not protective against COVID-19 contrary to findings by Ng et al. which showed adequate protection (28). However, an ideal mask for the prevention of the spread of SARS-CoV-2 is an area of current research. Our study reveals that majority of HCWs at Makerere University Teaching Hospitals have a negative attitude toward COVID-19 which is in congruence with a KAPs study on Ebola in Ethiopia among HCWs (27) but in contrast to Giao's study on COVID-19 (25). Only 44% of the HCWs in our study agreed that they could confidently participate in the management of patients with COVID-19 which implies that adequate information on COVID-19 case management should be provided to the HCWs. However, attitude was not significantly determined by knowledge.

Our study shows that HCWs in Makerere University Teaching Hospitals have good COVID-19 prevention practices similar to findings by Alfahan et al. on coronaviruses (29), Raab et al. on Ebola Virus Disease in Guinea (30) and in the general population of the Chinese on COVID-19 (19). Majority of the HCWs are following infection prevention and control practices recommended by the Ministry of Health Uganda and WHO. These include regular hand hygiene, social distancing and wearing a face mask when in high risk situations. Ninety-three percent and 96% of HCWs reported wearing a face mask when in contact with patients and washing hands before/after handling patients. These are very vital practices to prevent transfer of COVID-19 from patients to patients and to the HCWs themselves. However, up to 60% of HCWs admitted having avoided patients with symptoms suggestive of COVID-19. This can be attributed to shortage of personal protective equipment which has become a global problem (31–33).

Our study has some limitations. Firstly, no standardized tool for assessing KAPs on COVID-19 has been previously validated. We have however adapted and modified a previously published tool for assessment of KAP toward prevention of respiratory tract infections; and a tool used to assess KAP among Chinese residents (19, 20). The questions have been formulated from WHO and CDC guidelines and reports on COVID-19 (12). Secondly, only HCWs in Makerere University Teaching Hospitals were surveyed and the results of this study may not reflect the KAPs of HCWs in the entire country. However, this is the first study to assess KAPs, can be used to formulate targeted Continuing Medical Education (CME) for HCWs and enrolled in a countrywide survey and training on COVID. A similar study may be extended to the community. The study also had a low response rate (23%), which has been documented in web-based surveys especially among professionals (34) and this limits the survey's generalization.

In conclusion, we found that more than two-third of HCWs in Makerere University Teaching Hospitals have sufficient knowledge on the transmission, diagnosis and prevention of the transmission of COVID-19. Knowledge on COVID-19 was significantly higher among HCWs who used news media such as televisions and newspapers and those aged 18–39 were more knowledgeable about COVID-19. There was no statistically significant difference in the level of knowledge about COVID-19 among health care workers in Uganda irrespective of their professions or qualifications. About four-fifth of the respondents had poor attitude toward COVID-19 and just over 70% of the HCWs had good practices toward COVID-19 especially those aged 40 years or more. Continued professional education is advised among HCWs in Uganda to improve knowledge of HCWs hence averting negative attitudes and promoting positive preventive and therapeutic practices. We recommend follow up studies involving teaching and non-teaching hospitals across the country.

## Data Availability Statement

The datasets presented in this study can be found in online repositories. The names of the repository/repositories and accession number(s) can be found in the article/Supplementary Material.

## Ethics Statement

The proposal has been cleared by Mulago Hospital Research Ethics Committee (MHREC) Protocol Number MHREC 1866. The study was conducted according to the *Declaration of Helsinki* and all participants signed a written informed consent.

## Author Contributions

RO and FB designed the study protocol and analyzed the data and drafted the original manuscript. RO, FB, GC, GW, and DN participated in data collection. All authors reviewed and approved the final manuscript.

## Conflict of Interest

The authors declare that the research was conducted in the absence of any commercial or financial relationships that could be construed as a potential conflict of interest.

## Supplementary Material

***Questionnaire:***

doi: https://doi.org/10.6084/m9.figshare.12162045

***Dataset:***

doi: https://doi.org/10.6084/m9.figshare.12161910
