# Supplementary material for: Coronavirus Disease-2019: Knowledge, Attitude, and Practices of Health Care Workers at Makerere University Teaching Hospitals, Uganda
Source: Front Public Health. 2020 Apr 30;8:181. doi: 10.3389/fpubh.2020.00181 (PMC7204940; doi:10.3389/fpubh.2020.00181)
Supplement: Supplementary file 1 [file Data_Sheet_1.PDF]

COVID-KAP-HCW-Mulago Study

CORONAVIRUS DISEASE (COVID-19): KNOWLEDGE, ATTITUDE AND PRACTICES AMONG HEALTHCARE WORKERS AT MULAGO HOSPITAL.

*Required

Investigators

Olum RONALD (1)
Chekwech GAUDENCIA (1)
Wekha GODFREY (1)
Nassozi Dianah RHODA (1)
Bongomin Felix (2,3)

1. College of Health Sciences, Makerere University, Uganda.
2. Department of Internal Medicine, College of Health Sciences, Makerere University, Uganda.
3. Department of Medical Microbiology and Immunology, Faculty of Medicine, Gulu University, Uganda.

CLICK NEXT TO CONTINUE.

Consent Form

Coronavirus Disease 2019 also known as COVID-19 is rapidly expanding to a number of countries globally and is now a pandemic that has claimed over 30,000 lives. 33 cases have been confirmed in Uganda already as of 30th March 2020. Healthcare workers are at the forefront of the pandemic response and are exposed to dangers that put them at risk of acquiring COVID-19 infection. Hazards include pathogen exposure, long working hours, psychological distress, fatigue, occupational burnout, stigma, and physical and psychological violence. A number of healthcare workers have lost their lives to the disease already. A poor understanding of the disease among healthcare workers (HCWs) can result in delayed identification and treatment translating into rapid spread of infections and putting the patients' lives at risk.
A great number of media houses and websites are publishing unverified and false information on the disease. Healthcare workers must therefore be well equipped with the right information and possess a positive attitude towards COVID-19 and prevention practices. The purpose of the study is to assess the knowledge, attitude and practices of healthcare workers in Uganda towards COVID-19. Data from this study shall be used by the healthcare stakeholders to implement pandemic preparedness strategies. There is no direct benefit of the study to the participants.
Your personal details like emails, name, employee number, registration number and address are not required anywhere in this form.
Participating in the study is voluntary.
In case of any questions related to the study, please contact the Principal Investigator;

Olum Ronald
Email: olum.ronald@gmail.com
Tel: +256775512540

For any queries related to the study participant's rights, kindly address them to the Chairperson Mulago Hospital Research Ethics Committee,
Dr Frederick Nakwagala
Mobile: +256 772325869.
A detailed consent form can be downloaded from the link below;

https://drive.google.com/open?id=1lYtuh_3Zw-oHyRjleroUj1rI1_CR2Ge7

Statement of Consent

I have been asked to participate in the research study. The study has been explained to me. I understand what the study means to me including what I (the participant) have to go through while in the study. I have had an opportunity to ask questions about the study and have been answered in the best way for me to understand. If there are any other questions that I have to ask later, I will freely approach the study representatives whose contact I have been provided with. I also understand that my participation is voluntary and my consent can be withdrawn any time I wish to do so, without any penalty or loss of benefits to which I am otherwise entitled to.

BY CLICKING NEXT, I CONSENT TO TAKE PART IN THIS STUDY.

Demographics

1. Place of work. *

Mark only one oval.

- ( ) Mulago National Referral Hospital (Old Mulago)
- ( ) Kiruddu National Referral Hospital
- ( ) Kawempe National Referral Hospital
- ( ) Mulago Specialised Women and Neonatal Hospital.

2. Sex *

Mark only one oval.

- ( ) Female
- ( ) Male

3. Age in completed years *

4. Qualification *

Mark only one oval.

- Nurse
- Midwife
- Intern Doctor
- Medical Officer
- Senior House Officer
- Specialist

5. Highest level of education *

Mark only one oval.

- Certificate
- Diploma
- Bachelors
- Masters
- PhD

6. Source of information on COVID-19. Tick all that apply. *

Tick all that apply.

- [ ] Official international health organisation sites and media e.g. WHO, CDC.
- [ ] Official government sites and media e.g. Ministry of Health-Uganda.
- [ ] News Media e.g. TVs, radios, Magazines, Newspapers
- [ ] Social Media e.g. WhatsApp, Facebook, Twitter, Instagram
- [ ] Journals
- [ ] Others

Knowledge

7. The main clinical symptoms of COVID-19 are; (Tick all that apply) *

Tick all that apply.

- [ ] Fever
- [ ] Headache
- [ ] Myalgia (muscle pain)
- [ ] Smell disturbance
- [ ] Sore throat
- [ ] Runny nose
- [ ] Sneezing
- [ ] Diarrhoea
- [ ] Cough
- [ ] Confusion

8. There is currently no effective cure for COVID-19, but early symptomatic and supportive treatment can help most patients recover from the infection. *

Mark only one oval.

- True
- False
- I don't know

9. Not all persons with COVID-19 will develop severe cases. Only those who are elderly, have chronic illnesses, and are obese are more likely to be severe cases. *

Mark only one oval.

- True
- False
- I don't know

10. Eating or contacting wild animals would result in the infection by the COVID-19 virus. *

Mark only one oval.

- True
- False
- I don't know

11. Persons with COVID-2019 cannot transmit the virus to others when a fever is not present. *

Mark only one oval.

- True
- False
- I don't know

12. The COVID-19 virus spreads via respiratory droplets of infected individuals. *

Mark only one oval.

- True
- False
- I don't know

13. Wearing general medical masks can prevent one from acquiring infection by the COVID-19 virus. *

Mark only one oval.

- True
- False
- I don't know

14. It is not necessary for children and young adults to take measures to prevent the infection by the COVID-19 virus. *

Mark only one oval.

- True
- False
- I don't know

15. To prevent the infection by COVID-19, individuals should avoid going to crowded places such as bus parks and avoid taking public transportations. *

Mark only one oval.

- True
- False
- I don't know

16. Isolation and treatment of people who are infected with the COVID-19 virus are effective ways to reduce the spread of the virus. *

Mark only one oval.

- True
- False
- I don't know

17. People who have contact with someone infected with the COVID-19 virus should be immediately isolated in a proper place. In general, the observation period is 14 days. *

Mark only one oval.

- True
- False
- I don't know

Attitude

18. Black race is protective towards COVID-19 disease. *

Mark only one oval.

- Strongly disagree
- Disagree
- Not sure
- Agree
- Strongly agree

19. Wearing a well-fitting face mask is effective in preventing COVID-19. *

Mark only one oval.

- Strongly disagree
- Disagree
- Not sure
- Agree
- Strongly agree

20. Using a hand wash can prevent you from getting COVID-19. *

Mark only one oval.

- Strongly disagree
- Disagree
- Not sure
- Agree
- Strongly agree

21. When a patient has signs and symptoms of COVID-19, I can confidently participate in the management of the patient. *

Mark only one oval.

- Strongly disagree
- Disagree
- Not sure
- Agree
- Strongly agree

22. Uganda is in a good position to contain COVID-19. *

Mark only one oval.

- Strongly disagree
- Disagree
- Not sure
- Agree
- Strongly agree

Practices

23. In recent days, I have gone to any crowded place? *

Mark only one oval.

- Always
- Occasional
- Never

24. In recent days, I have worn a mask when in contact with patients? *

Mark only one oval.

- Always
- Occasional
- Never

25. In the recent days, I have refrained from shaking hands. *

Mark only one oval.

- Always
- Occasional
- Never

26. In the recent days, I have washed my hands before and after handling each patient? *

Mark only one oval.

- Always
- Occasional
- Never

27. In the recent days, I have avoided patients with signs and symptoms suggestive of COVID-19. *

Mark only one oval.

- Always
- Occasional
- Never

This content is neither created nor endorsed by Google.
